# Patients With Severe Alcohol-Related Cognitive Impairment Improve in Flexibility When Abstinence Is Maintained: A Comparative Study With Alzheimer’s Disease

**DOI:** 10.3389/fpsyg.2022.936639

**Published:** 2022-07-01

**Authors:** Virgile Clergue-Duval, Thomas Barré, Emmanuel Cognat, Anne-Laure Brichet, Claire Géraud, Julien Azuar, Philippe Michaud, Dorothée Lecallier, Sonia Arfaoui-Geffroy, Eric Hispard, Claire Paquet, Frank Bellivier, Frank Questel, Florence Vorspan, all members of the ResAlCog care network

**Affiliations:** ^1^Département de Psychiatrie et de Médecine Addictologique, Site Lariboisière Fernand-Widal, GHU APHP. Nord - Université Paris Cité, APHP, Paris, France; ^2^ResAlCog (Réseau Pour la Prise en Charge des Troubles Cognitifs Liés à L’alcool), Paris, France; ^3^Inserm UMRS-1144, Optimisation Thérapeutique en Neuropsychopharmacologie, Université Paris Cité, Paris, France; ^4^UFR de Médecine, Université Paris Cité, Paris, France; ^5^Centre de Neurologie Cognitive, Site Lariboisière Fernand-Widal, GHU APHP. Nord - Université Paris Cité, APHP, Paris, France; ^6^Clinique des Epinettes, Paris, France; ^7^Unité Serge Korsakoff-Maison d’Accueil Spécialisée, Villeneuve-la-Garenne, France; ^8^Clinique Médicale du Parc, Saint-Ouen-l'Aumône, France

**Keywords:** alcohol-related cognitive impairment, alcohol brain damage, alcohol use disorder, Alzheimer’s disease, cognitive impairment, disease progression

## Abstract

The disease progression of severe alcohol-related cognitive impairment (ARCI) is debated. The aim of this study was to compare the cognitive change of patients with severe ARCI in inpatient setting to that of patients with Alzheimer’s disease (AD). Fifteen consecutive patients with severe ARCI were recruited between 2013 and 2015. They received inpatient detoxification, neurological assessment, and inpatient cognitive rehabilitation in specialized facilities. Twelve patients, with documented AD matched on sex and initial cognitive impairment severity, were selected. All have benefited from two neuropsychological assessments. The neurocognitive change was tested in both groups with pair-wised Wilcoxon tests. ARCI and AD patients’ time course was compared with Mann–Whitney–Wilcoxon test. In ARCI group, first assessment occurred at 2.9 (± 2.2) months of abstinence and follow-up 6.5 (± 2.9) months later, the mean age was 56.5 (± 7.4) years, and 12 were men. In AD group, follow-up occurred at 12.8 (± 2.9) months (*p* < 10–3), the mean age was 72.3 (± 8.4) years (*p* < 10–3), and 10 were men. ARCI patients significantly improved on one executive function test (TMT-B; *p* < 0.05), while AD patients have worsened memory subtests on Free-and-Cued-Selective-Reminding Test (*p* < 0.05). These tests showed a statistically different change between severe ARCI and AD group (*p* < 0.05). Severe ARCI patients have improved in executive functioning, discernible on the TMT-B test, in specific care setting, including abstinence maintenance and rehabilitation. The disease progression was different from that observed in AD patients.

## Introduction

In the general population, alcohol use disorder is the strongest modifiable risk factor for dementia onset and the primary risk factor for early dementia (Schwarzinger et al., 2018). The development of intensive cognitive rehabilitation therapy with prolonged abstinence has been demonstrated to significantly improve alcohol-related cognitive impairments (ARCI) in non-amnesic and non-demented patients (Rupp et al., 2012; Bates et al., 2013; Maillard et al., 2020). Severe ARCI (formerly labeled “alcoholic dementia”) and Wernicke–Korsakoff syndrome have been initially described as an acquired irreversible condition. However, the prognosis of the disorder, especially with the development of new intensive cognitive rehabilitation programs, is still debated. Some authors proposed a specific and tailored cognitive rehabilitation training associated with alcohol abstinence. Those rehabilitation programs were diverse but included, for example, motoric procedural learning by the repetition of simple motor tasks, cognitive procedural learning, and memory training (including the repetition of laws and algorithms; Oudman et al., 2015), repetitive memory training (Kashima et al., 1999), and intensive errorless training with the support of external reminders, calendars, and alarms (Kopelman et al., 2009; Svanberg and Evans, 2013). Furthermore, the adjunction of behavioral therapy reinforcement of the memory training, and the rule provision in executive tasks, improved the performances (Svanberg and Evans, 2013). Most of the evidence of improvement abilities in patients diagnosed with Wernicke–Korsakoff syndrome or severe ARCI come from comparative studies comparing the learning abilities of patients with Wernicke–Korsakoff syndrome with those of healthy controls on one test at a time (Svanberg and Evans, 2013; Oudman et al., 2015). Those studies have limited sample sizes (ranging from single case report to 5 to 10 patients compared to matched controls). Very few studies prospectively assessed the global functioning of patients before and after a comprehensive cognitive rehabilitation program. To date, despite the demonstrated abilities of each rehabilitation exercise to improve one or the other cognitive function assessed by a specific test, the transferability of those results in a global improvement of patient functioning is not clearly demonstrated.

Among the few prospective data on the change of cognitive impairment in Wernicke–Korsakoff syndrome or severe ARCI, two studies deserve a specific attention (Fujiwara et al., 2008; Maillard et al., 2021). Regarding the episodic memory impairment, both Fujiwara et al. (*n* = 20) and Maillard et al. (*n* = 8) showed the stability of impairment (Fujiwara et al., 2008; Maillard et al., 2021). Regarding the executive functions, the results were discordant and only Fujiwara et al. showed a possible improvement in some of the executive functions at follow-up after 2 years, but the patients were abstinent since several years at baseline (Fujiwara et al., 2008). This wide variability may be related to the heterogeneity in patients included as having Wernicke–Korsakoff syndrome, already a difficult classification (Schwarzinger et al., 2018; Azuar et al., 2021), as well as the heterogeneity of assessment in terms of choice in neurocognitive tests, of time since the diagnosis or time between the test and the retest.

In order to participate in this debate, we choose to study the cognitive change of patients with established severe ARCI, where the initial evaluation was made after the elimination of all curable and easy to improve causes of cognitive deficit, and where all the differential diagnoses have been ruled out. All those patients were referred to a specialized addiction medicine ward for diagnosis. Furthermore, they all received intensive long lasting inpatient cognitive rehabilitation in specialized facilities using the most updated rehabilitation procedures. As alcohol abstinence was maintained in those care facilities, we can confidently rule out alcohol relapse as a cause of heterogeneity in the cognitive change.

The aims of this study were to observe the cognitive change of those patients with severe ARCI supported in specialized facilities in cognitive rehabilitation and to compare to the disease progression of patients with established Alzheimer’s disease matched on sex and initial cognitive impairment.

## Materials and Methods

### Subjects

#### Severe Alcohol-Related Cognitive Impairments Group

For this comparative chart review, study patients with severe ARCI were included. Patients were recruited in the addiction medicine ward of Parisian university hospital. All consecutive patients were included during 2 years between 2013 and 2015. Patients were screened after at least 1 month of inpatient detoxification using the Montreal Cognitive Assessment, with a threshold below 25 points (Nasreddine et al., 2005). They were then undergoing a full neuropsychological testing. Based on previous studies (Caine et al., 1997; Pitel et al., 2011; Sachdev et al., 2014), patients with severe ARCI were precisely defined as presenting with the three following criteria. First, the patient has to present at least two different impaired cognitive functions on the first neuropsychological assessment, including executive functions and episodic memory. Second, the patient had to present a retrospective documented clinical sign evocative of Wernicke’s encephalopathy such as malnourishment and/or a neurologic sign (ataxia or eye movement’s disorder). Malnourishment itself was defined as either weight loss ≥ 10%, with respect to any previously recorded weight from a previous medical record; or weight loss ≥ 5% in 1 month, with respect to a previously recorded weight; or Body Mass Index ≤ 17 kg/m^2^, or serum albumin < 30 g/l; or serum prealbumin < 110 mg/l. Third, any other documented etiology for dementia (degenerative or vascular) had to be ruled out with the adequate brain imaging and/or cerebrospinal fluid examination (Azuar et al., 2021).

All patients diagnosed with severe alcohol-related neurocognitive disorder were then referred to specific inpatient long lasting residential rehabilitation programs (all members of the ResAlCog care network). Cognitive rehabilitation program included patient stimulation in everyday life by all members of the healing staff and specific sessions of memory and executive functions rehabilitation provided by trained neuropsychologists.

Using these criteria, 31 patients with severe ARCI were selected between 2013 and 2015. Among them, 15 patients had a documented second standardized neuropsychological assessment allowing the description of a time change.

#### Alzheimer’s Disease Group

To constitute a control group, 12 patients were selected from Expert Center on Cognitive disorders located in the same hospital. All were diagnosed with mild cognitive impairment (MCI) due to Alzheimer’s disease (Albert et al., 2011). The two groups matched on sex and the initial level of cognitive impairment at the time of diagnosis according to screening tools. As patients from this outpatient program are evaluated with the MMSE (Folstein et al., 1975), the previously published correspondence between the Montreal Cognitive Assessment and MMSE (Trzepacz et al., 2015) was used to match the two groups.

### Neurocognitive Assessment

Standardized test assessing executive functions, episodic memory, and visuo-spatial ability that are known to be impaired in patients with ARCI (Ihara et al., 2000; Pitel et al., 2008, 2009; Stavro et al., 2013; Le Berre et al., 2017) and also available in patients AD group was selected.

Executive functions were assessed with the Verbal fluency task (Cardebat et al., 1990), including a letter and a category fluency tasks. Flexibility was also assessed with the Trail Making Test (TMT-B) expressed as errors and speed (Reitan, 1955). To assess a processing speed as well as to adjust the patients on premorbid educational level, the Wechsler Adult Intelligence Scale III or IV (Saklofske and Schoenberg, 2011) was used, especially the coding subtest. Verbal conceptualization was assessed with the similarities subtest from the WAIS III or IV. Memory was assessed through with two different tests. The 16-item Free-and-Cued Recall, a French validated version of the free-and-cued-selective reminding test (FCSRT) (Van der Linden et al., 2004), was used for verbal and anterograde memory assessment. The Rey Complex Figure Test Copy (McKinlay, 2011), expressed in score and speed, was used for the Visuo-spatial processing.

### Statistical Analysis

The comparability of the two groups was tested with Chi-squared test for sex and Mann–Whitney–Wilcoxon test for age, initial cognitive impairment, and time elapsed between the two standardized neuropsychological testing.

In each group, pair-wised Wilcoxon tests were performed to test for a time effect between the two available standardized neuropsychological testing for all scores and speeds that were expressed as continuous variables. Chi-squared test was used for scores expressed as categories, such as Rey’s complex figure copy classification. Furthermore, the delta score between the two standardized neuropsychological testing was compared between severe ARCI and AD groups with Mann–Whitney–Wilcoxon test. The alpha risk was set at 0.05. All statistical analyses were performed with R software version 3.2 and SPSS software version 21.0.

## Results

### Characteristics of Patients

For patients diagnosed with severe ARCI, 12 were men (80%), the mean age was 56.6 years (SD ± 7.4), the alcohol use disorder duration mean was 22 years (± 10), the first neuropsychological assessment occurred at 2.8 (± 2.1) months of abstinence, and the second assessment was performed at 6.5 (± 2.9) months after the first one. The clinical characteristics of patients with severe ARCI are provided in Table 1. The patients in ARCI group were younger than those in AD group (72.2 years (± 8.4); *p* < 0.001) and the average time elapsed between the two standardized neuropsychological testing was longer in AD group [12.8 months (±2.9); *p* < 0.001; Table 2]. There were no differences between the groups in sex (*p* = 0.83) or severity of the cognitive impairment (*p* = 0.93).

**Table 1:** Sociodemographic and clinical characteristics of the patients in the severe alcohol-related cognitive impairment group (*N* = 15) [Number (percentage) or mean (SD; minimum–maximum)].

	*N* (%)
Male	12 (80%)
More than High School diploma	7 (46.7%)
No education or less than High School	8 (53.3%)
Sign of malnourishment^†^	6 (40%)
Not available	1 (6.7%)
Liver cirrhosis	6 (40%)
Lower limbs neuropathy	6 (40%)
Past history of epilepsy	4 (26.7%)
	Mean (SD) [Min–Max]
Age (years)	56.6 (±7.4) [46–68]
Body Mass Index (BMI; kg/m^2^)	22.4 (±4) [16–31]
Alcohol use disorder duration (years)	22 (±10) [5–34]
Time between baseline and follow-up assessment (months)	6.5 (±2.9) [2.3–13.2]
Length of abstinence before baseline assessment (months)	2.8 (±2.1) [0.3–7.6]

† *Defined as either: (i) Weight loss ≥ 10%, with respect to any previously recorded weight from a previous medical record. (ii) Weight loss ≥ 5% in 1 month, with respect to a previously recorded weight. (iii) BMI ≤ 17 kg/m^2^. (iv) Serum albumin < 30 g/L. (v) Serum prealbumin < 110 mg/L*.

**Table 2:** Demographic and cognitive assessments characteristics in severe alcohol-related cognitive impairment (severe ARCI) group and Alzheimer’s disease (AD) group.

	Severe ARCI	AD	Test
Male (Number, %)	12 (80%)	10 (83%)	*χ*^2^ = 0.49 *p* = 0.83
Age (years) Mean (± SD). [Min-Max]	56.6 (± 7.4) [46–68]	72.2 ± 8.4 [57–87]	MWW U = 14.500 *p* < 0.001
Time between baseline and follow-up assessment (months) Mean (± SD). [Min-Max]	6.5 (± 2.9) [2.3–13.2]	12.8 ± 2.9 [8–20]	MWW U = 13.000 *p* < 0.001
MoCA (expressed in MMSE equivalent) or MMSE (*N* = 12) Mean (±SD)	24.3 (± 2.8)	24.2 (± 3.1)	MWW U = 70.500 *p* = 0.93

*SD, standard deviation. MoCA, Montreal cognitive assessment. MMSE, mini-mental state examination. MWW, Mann–Whitney–Wilcoxon test*.

### Cognitive Change

**Figure 1:**
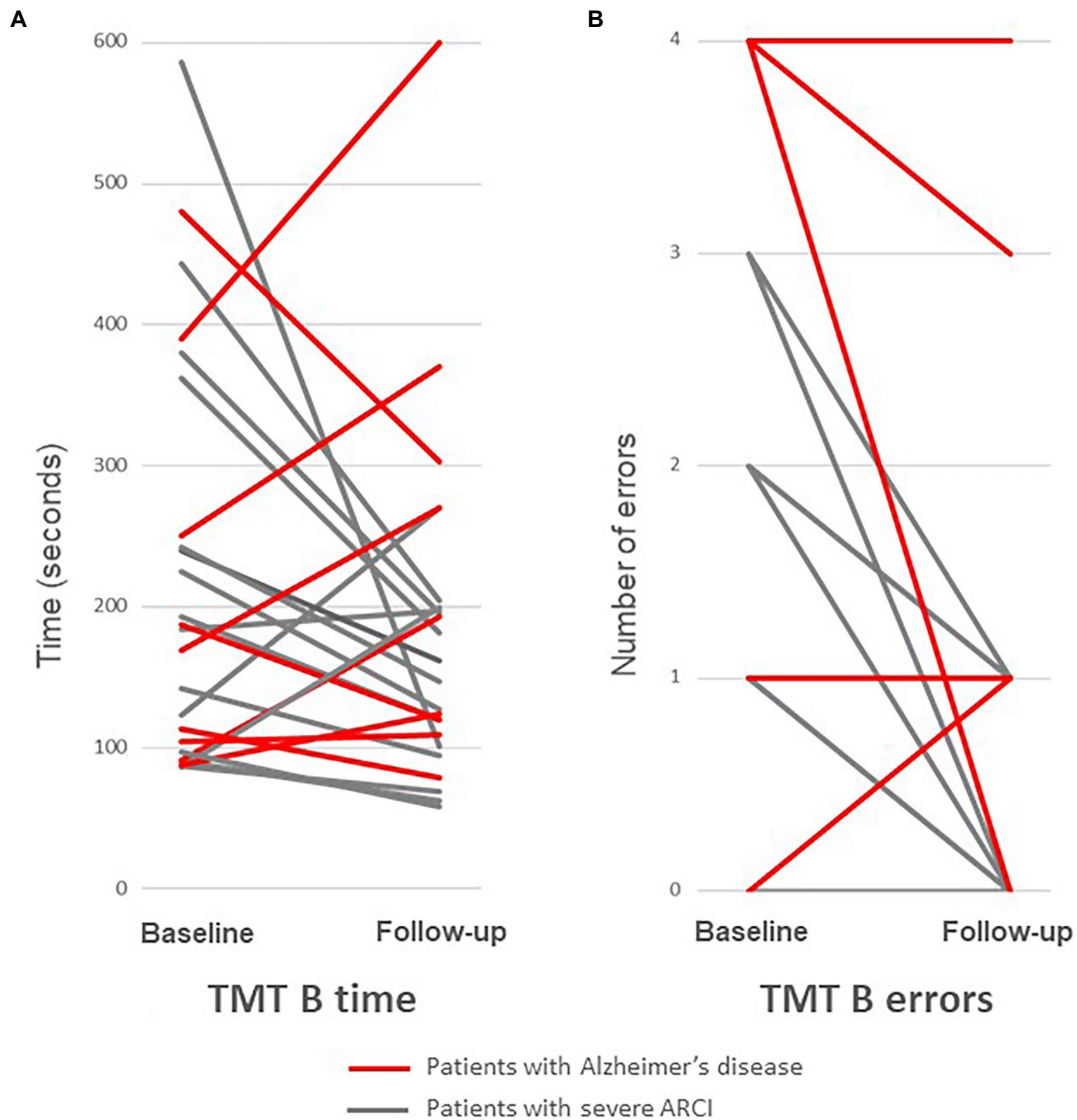
Change in TMT-B test between baseline and follow-up in severe Alcohol-Related Cognitive Impairment (severe ARCI) group (*n* = 13; in gray) and in Alzheimer’s disease group (*n* = 10; in red): TMT-B time **(A)** and TMT-B errors **(B)**. Severe ARCI patients improve between baseline and follow-up (TMT-B time: *p* = 0.013 and TMT-B errors: *p* = 0.016, in Pair-wised Wilcoxon test), while AD patients were stable.

Patients with severe ARCI did significantly improve on two measures of executive functioning collected by the TMT-B test: errors (*p* = 0.016) and speed (*p* = 0.013; Table 3; Figure 1). Furthermore, it was observed a significant improvement in Rey complex figure copy classification (*p* = 0.049) in severe ARCI group. Indeed, 3 patients improved (construction type evolved from V to III, another one from V to IV, and one normalized from IV to I), 7 patients were stable, 1 patient worsened (from II to IV), among the 11 patients with data available on this test. No significant changes were observed in verbal fluency task, in WAIS, in FCSRT memory tests, and in Rey complex figure score and time (*p* > 0.05; Tables 3-5).

**Table 3:** Results of cognitive tests of executive functions at baseline (B) and follow-up (F) in severe Alcohol-Related Cognitive Impairment (severe ARCI) group (*n* = 15) and in Alzheimer’s disease group (*n* = 12).

	Severe ARCI group				Alzheimer’s disease group				Comparison of F–B delta score between groups^‡^
	*n*	Baseline (B)	Follow-up (F)	Test between F and B^†^	*n*	Baseline (B)	Follow-up (F)	Test between F and B^†^	
Letter fluency tasks	14	14.13 ± 4.96	15.79 ± 7.20	*Z* = –0.942 *p* = 0.346	11	20.91 ± 11.37	19.45 ± 8.82	*Z* = –0.981 *p* = 0.327	*p* = 0.217
Category fluency tasks	14	16.87±6.94	16.07 ± 7.21	*Z* = –0.157 *p* = 0.875	11	19.73 ± 7.00	18.91 ± 7.56	*Z* = –0.627 *p* = 0.531	*p* = 0.783
TMT-B time (seconds)	13	242.4 ± 155.7	146.9 ± 65.1	Z = −2.481 p = 0.013^*^	10	195.8 ± 138.3	236.8 ± 158.5	*Z* = –1.274 *p* = 0.203	*p* = 0.0147
TMT-B errors	13	1.62±1.19	0.73 ± 1.03	Z = –2.414 p = 0.016^*^	10	1.50 ± 1.78	1.10 ± 1.37	*Z* = –0.816 *p* = 0.414	*p* = 0.121
WAIS III or IV. Coding	10	40.08±12.94	41.36 ± 14.74	*Z* = –1.480 *p* = 0.139	3	34.33 ± 5.13	26.0 ± 15.0	*Z* = –1.069 *p* = 0.285	*p* = 0.0883
WAIS III or IV. Similarities	7	17.13±4.42	16.86 ± 6.93	*Z* = –0.339 *p* = 0.734	0	–	–	–	–

* *Significant improvement*.

† *Pair-wised Wilcoxon test*.

‡ *Mann–Whitney–Wilcoxon test*.

*Severe ARCI patients improve between baseline and follow-up in TMT -B, while AD patients were stable*.

**Table 4:** Results of cognitive tests of memory at baseline (B) and follow-up (F) in severe Alcohol-Related Cognitive Impairment (severe ARCI) group (*n* = 15) and in Alzheimer’s disease group (*n* = 12).

	Severe ARCI group				Alzheimer’s disease group				Comparison of F–B delta score between groups^‡^
	*n*	Baseline (B)	Follow-up (F)	Test between F and B^†^	*n*	Baseline (B)	Follow-up (F)	Test between F and B^†^	
FCSRT. Total free recall	14	11.20±9.01	14.79 ± 8.93	*Z* = –1.455 *p* = 0.146	12	13.50 ± 9.33	10.75 ± 8.99	Z = –2.199 p = 0.028^§^	p = 0.0231
FCSRT. Total free-and-cued recall	14	30.00±10.96	35.2 ± 9.04	Z = –1.414 *p* = 0.157	12	31.50 ± 13.44	25.50 ± 16.16	Z = –2.505 p = 0.012^§^	p = 0.009175
FCSRT. Cue reactivity	14	54.13±21.89	64.99 ± 19.78	*Z* = –1.319 *p* = 0.187	9	58.80 ± 25.29	45.47 ± 28.96	Z = –2.666 p = 0.008^§^	p = 0.0128
FCSRT. Recognition	14	12.40±3.83	13.29 ± 4.69	*Z* = –1.268 *p* = 0.205	11	14.73 ± 1.48	14.00 ± 3.19	*Z* = –0.562 *p* = 0.574	*p* = 0.417
FCSRT. False recognition	14	0.8±1.06	0.57 ± 0.93	*Z* = −0.785 *p* = 0.432	11	0.82 ± 1.53	0.64 ± 1.02	*Z* = –0.447 *p* = 0.655	*p* = 0.468
FCSRT. Free delayed recall	14	3.00±3.76	3.57 ± 4.071	*Z* = –0.599 *p* = 0.549	11	5.18 ± 3.65	4.55 ± 4.32	*Z* = –0.496 *p* = 0.620	*p* = 0.393
FCSRT. Free-and-cued total delayed recall	14	9.67±4.73	11.57 ± 4.51	*Z* = –0.875 *p* = 0.381	11	11.73 ± 4.49	9.36 ± 6.15	Z = –2.442 p = 0.015^§^	p = 0.0298
FCSRT. Intrusions. Raw score	13	23.67±20.98	15.23 ± 16.20	*Z* = –1.363 *p* = 0.173	12	7.00 ± 6.35	10.00 ± 6.79	*Z* = –1.572 *p* = 0.116	*p* = 0.108
Rey Figure. Immediate recall	5	4.40±4.96	6.00 ± 4.47	*Z* = –1.604 *p* = 0.109	8	10.50 ± 5.81	11.56 ± 6.08	*Z* = –1.262 *p* = 0.207	p = 0.0333

§ *Significant worsening*.

† *Pair-wised Wilcoxon test*.

‡ *Mann–Whitney–Wilcoxon test*.

*Severe ARCI patients were stable, while AD patients worsened*.

In contrast, in AD patients, it was observed a significant worsening in several FCSRT memory subtests: FCSRT Total free recall (*p* = 0.028), Total free-and-cued recall (*p* = 0.012), Cue reactivity (*p* = 0.008), and Free-and-cued total delayed recall (*p* = 0.015). No significant changes were observed in the executive tests (TMT-B, verbal fluency test and WAIS) and in the Rey complex figure test (*p* > 0.05; Tables 3 and 4).

**Table 5:** Results of cognitive tests of visuo-spatial abilities at baseline (B) and follow-up (F) in severe Alcohol-Related Cognitive Impairment (severe ARCI) group (*n* = 15) and in Alzheimer’s disease group (*n* = 12).

	Severe ARCI group				Alzheimer’s disease group				Comparison of F–B delta score between groups^‡^
	*n*	Baseline (B)	Follow-up (F)	Test between F and B^†^	*n*	Baseline (B)	Follow-up (F)	Test between F and B^†^	
Rey Figure copy. Time (minutes)	8	5.27±2.90	4.37 ± 2.06	*Z* = –1.120 *p* = 0.263	12	4.30 ± 2.21	3.30 ± 2.31	*Z* = –1.020 *p* = 0.308	*p* = 0.967
Rey Figure copy. Score	11	31.23±3.25	31.91 ± 5.55	*Z* = –1.809 *p* = 0.071	12	30.00 ± 3.10	26.29 ± 11.39	*Z* = –0.511 *p* = 0.609	*p* = 0.207

† *Pair-wised Wilcoxon test*.

‡ *Mann–Whitney–Wilcoxon test*.

*Severe ARCI patients and AD patients were stable*.

### Disease Progression Between Severe ARCI and AD

Regarding the disease progression, in comparison of cognitive changes between severe ARCI and AD, it was observed a statistically difference in TMT-B time (*p* = 0.015), immediate recall score of Rey figure (*p* = 0.033), and several FCSRT memory subtests: FCSRT Total free recall (*p* = 0.023), Total free-and-cued recall (*p* = 9.2 × 10^-3^), Cue reactivity (*p* = 0.013), and Free-and-cued total delayed recall (*p* = 0.030; Table 3; Figure 1).

Two years later, the last clinical information available for the 15 patients with severe ARCI was that eight were still in specialized long-term rehabilitation facilities, where they will stay for several years. Three patients were in a nursing home where they will remain for their lifetime. Two patients were still hospitalized in an acute ward and were on waiting list for nursing home placement. Two patients had a clinically relevant improvement in terms of functional status that could qualify for “rehabilitation.” One of them could be discharged and go back home. Another one had been discharged and was homeless.

## Discussion

In this study, whose objectives were to observe the cognitive change in 15 patients with severe ARCI in comparison with 12 AD patients matched on sex and initial cognitive impairment, we observed a different disease progression between the two groups. In 15 severe ARCI patients, it was not observed a cognitive worsening of memory function at 6.5 months of follow-up, contrarily to AD patients. Moreover, in the very stringent severe ARCI group constituted to reduce clinical heterogeneity by having eliminated the others etiologies for dementia (Azuar et al., 2021) and where alcohol abstinence was documented and where continuous neuropsychological rehabilitation was delivered during a 9 month follow-up on average, it was observed a modest but statistically significant improvement on executive functions, discernible on the TMT-B test. The disease progression was different between severe ARCI and AD patients. Although most severe ARCI patients (all but two) remained severely impaired and could not be discharged, these results could demonstrate at least the absence of degenerative disease progression of the cognitive deficit, and even sensitivity to time and/or alcohol abstinence maintenance and/or cognitive rehabilitation programs.

Regarding the time needed to observe a cognitive improvement, in patients with alcohol use disorder and moderate cognitive impairment, screened immediately after alcohol cessation, Stravo et al. showed that moderate cognitive impairment remained stable during the first year of alcohol abstinence (Stavro et al., 2013). On the other hand, Pitel et al. recommended a 6 month abstinence period before retest to observe potential improvement (Pitel et al., 2009). In patients with Wernicke–Korsakoff syndrome, the improvement in executive function observed by Fujiwara et al. occurred after 2 years in specific remediation, in patients previously abstinent for several years (Fujiwara et al., 2008). Maillard et al. chose a 1-year period before the retest (Maillard et al., 2021). Compared to the latter two, the time of follow-up in this study, with a second assessment at 6.5 month after baseline, may have been too short to capture the comprehensive effect of both abstinence and rehabilitation. Nevertheless, these results show a discernible improvement in TMT-B test after this period, underlining the relevance of performing follow-up assessments as early as 6 months. TMT-B test could be a useful recovery marker, which had not been studied in the two previous studies (Fujiwara et al., 2008; Maillard et al., 2021). Of note, in populations of subjects without AUD and under the acute effect of alcohol with a blood alcohol concentration ≤ 1 mg/ml, Jongen et al. showed the absence of alteration of the TMT-B results (Jongen et al., 2016). This fact reinforces the specificity of TMT-B as a reversible marker of severe alcohol-induced executive function impairment rather than a marker of acute alcohol intoxication on cognitive abilities.

Strengths of this study were to study a hard to reach, relatively homogeneous population by carefully ruling out differential diagnoses, and the use of standardized neuropsychological testing allowed comparison, even with patients visiting a specialized AD center. Lastly, because patients with severe ARCI were maintained in specialized settings dedicated to cognitive rehabilitation belonging to the ResAlCog care network, it is possible to confidently state that the 15 patients described here maintained alcohol abstinence over time.

However, there were several limitations. First, there is not control group with healthy subjects to estimate effects of repeated testing or age-related decrease. Second, there is not control group with severe ARCI patients but without cognitive rehabilitation. Third, the number of patients is low although comparable to the only two previous published studies that we could identify. Fourth, some cognitive functions were not evaluated. For example, inhibition, evaluated with the Stroop test, was not available. Fourth, there is a possible reliability bias in data recorded, for example, regarding alcohol use in patients visiting the AD center. Fifth, the length between the two neuropsychological testing was not strictly comparable, between severe ARCI and AD patients. Lastly, we are not able to reliably associate the improvement in the result of this executive function test and the improvement observed for some patients regarding autonomy in everyday tasks.

This study opens three development perspectives. In terms of conceptualization this study clearly supports that even severely affected, ARCI patients have a different disease evolution pattern than patients affected by a neurodegenerative disorder. In terms of research on ARCI, future studies’ design should include multimodal prospective assessments of executive functions, including validated tests but also scales of autonomy in everyday tasks, above the classical various memory tests. In terms of care provided for those patients, as an improvement in the executive tests is observable as soon as 6 months, residential setting combining alcohol-free environment and specific cognitive rehabilitation programs should be proposed as soon as the diagnosis is confirmed for at least 6 months.

## Conclusion

This study provides a new long-term course description of patients with severe ARCI who received optimal medical diagnosis, up-to-date cognitive rehabilitation, in a care setting where alcohol abstinence can be monitored. We observed an average partial improvement on executive functions, and two patients out of 15 had a clinically relevant functional improvement. Conversely to patients with AD selected to have a comparable initial cognitive impairment, no neuropsychological worsening was shown in severe ARCI patients, especially on memory tests. Among executive functions studied, flexibility and visuo-spatial planning improved. Studies based on larger samples followed for a longer period of time to confirm these data may be helpful.

## Data Availability Statement

The datasets presented in this article are not readily available because it is the property of the ResAlCog network’s structures. Requests to access the datasets should be directed to FQ, franck.questel@aphp.fr.

## Ethics Statement

This study was conducted in adherence to the guidelines of Declaration of Helsinki and following French laws on biomedical research (Loi Jardé 2014, décrets d’application 2017). Our hospital has obtained a specific authorisation for the analysis of anonymized data collected during routine care, delivered by the French national board for information systems and freedom (Commission Nationale Informatique et Liberté; Number 2017–013). This chart review study design was approved by an internal ethics committee. Patients and their representatives were informed and could object to the use of their anonymized routine health care data for research purposes. Written informed consent for participation was not required for this study in accordance with the national legislation and the institutional requirements.

## Author Contributions

FQ, FV, and EC designed the study. TB, EC, and FQ collected the data. VC-D, TB, and FV worked on data analyses. VC-D, TB, and FV drafted the manuscript, with support of EC and FQ. FQ and FV supervised the study. All authors contributed to the article and approved the submitted version.

## Conflict of Interest

FV had congress fees paid by CAMURUS AB, RECORDATI, and ACCORD Pharmaceutical. FV gave a single lecture for RECORDATI (2020) and for CAMURUS AB (2022) and served in advisory boards for CAMURUS AB (2019, 2022), and ACCORD HEALTH CARE (2021). All payments were made to a research entity and not directly to FV. CP is a member of the International Advisory Boards of Lilly, is a consultant with Fujiribio, Alzohis, Neuroimmune, and Gilead, and is involved as an investigator in several clinical trials for Roche, Esai, Lilly, Biogen, Astra-Zeneca, Lundbeck, and Neuroimmune, outside the submitted work. EC is involved as an investigator in several clinical trials for Roche, Esai, and Biogen, outside the submitted work.

The remaining authors declare that the research was conducted in the absence of any commercial or financial relationships that could be construed as a potential conflict of interest.

## Publisher’s Note

All claims expressed in this article are solely those of the authors and do not necessarily represent those of their affiliated organizations, or those of the publisher, the editors and the reviewers. Any product that may be evaluated in this article, or claim that may be made by its manufacturer, is not guaranteed or endorsed by the publisher.
